# Novel ABCB4 mutation in a female patient with progressive familial intrahepatic cholestasis type 3: a case report and literature review

**DOI:** 10.1097/MS9.0000000000002813

**Published:** 2024-12-19

**Authors:** Israa Sharabati, Ruaa Mustafa Qafesha, Mohamed M.M. Mustafa, Mahmoud Diaa Hindawi, Heba Rasras, Sami Bannoura, Mohammed Abdulrazzak, Ibrahim Shamasneh

**Affiliations:** a Faculty of medicine, Al-Quds University, Jerusalem, Palestine; b Medical research group of Egypt, Negida Academy, Arlington, MA, USA; c Faculty of Pharmacy, Egyptian Russian University, Badr City, Egypt; d Faculty of Medicine, Al-Azhar University, Cairo, Egypt; e Department of Pathology, Al-Ahli Hospital, Hebron, Palestine; f Faculty of Medicine, University of Aleppo, Aleppo, Syria; g Department of Pediatrics, Al-Ahli Hospital, Hebron, Palestine

**Keywords:** ATP binding cassette subfamily B member 4 (ABCB4) gene, case report, multidrug-resistant protein 3 (MDR3), progressive familial intrahepatic cholestasis type 3

## Abstract

**Introduction and importance:**

Progressive familial intrahepatic cholestasis (PFIC) is an uncommon disorder inherited in an autosomal recessive manner. PFIC type 3 (PFIC-3) results from mutations in the ABCB4 gene. This type typically advances from chronic cholestasis, which may occur with or without jaundice.

**Case presentation:**

A 16-year-old female presented with abdominal pain, later developing liver complications. Genetic testing revealed a novel ABCB4 gene mutation linked to cholestasis. Diagnosed with PFIC-3, she was treated with ursodeoxycholic acid (UDCA) and vitamins, leading to improved liver function. Despite uncertain clinical significance of the mutation, predictions suggested it was damaging. Her liver function fully recovered, and she remained in remission during follow-up visits.

**Clinical discussion:**

PFIC3 is a rare, autosomal recessive disorder causing cholestasis and liver damage. Our study reported a young female with a novel ABCB4 mutation who responded well to UDCA. Diagnosis relies on comprehensive evaluation, and treatment options include UDCA, surgery, and liver transplantation.

**Conclusion:**

PFIC-3 gene must be considered while evaluating a young female with symptoms of cholestasis.

## Introduction and importance

Progressive familial intrahepatic cholestasis (PFIC) refers to a group of rare, inherited autosomal recessive liver diseases primarily affecting infants, young children, and adolescents. Six types of PFIC have been identified, classified based on specific gene mutations, including the traditional types 1, 2, and 3^[1]^. PFIC impairs hepatocyte bile secretion due to alterations in the proteins involved in this process.

A mutation in the ABCB4 gene causes PFIC-3, which affects the encoding of the multidrug resistance protein 3 (MDR3). MDR3 is a phosphatidylcholine transporter located on the canalicular membrane of hepatocytes. It plays a crucial role in emulsifying bile salts and protecting liver and bile duct epithelial cells from bile salt damage. Homozygous or compound heterozygous mutations in ABCB4 are characteristic of PFIC-3^[2]^.

Cholestasis, manifesting as jaundice and pruritus, is the hallmark clinical feature of PFIC. In PFIC-1 and PFIC-2, symptoms often appear early in infancy or the neonatal period. By contrast, PFIC-3 typically presents later in childhood or adulthood, with cirrhosis or portal hypertension sometimes being the first clinical signs^[1,3]^. In females, hormonal changes may lead to misdiagnosis, with PFIC-3 symptoms confused with contraceptive-induced cholestasis or intrahepatic cholestasis of pregnancy (ICP)^[4]^. Additionally, PFIC-3 is characterized by elevated gamma-glutamyl transferase (GGT) levels^[3]^.

The diagnosis of PFIC is typically made by excluding other cholestatic diseases through initial laboratory assessments, imaging, liver pathology, and, finally, genetic testing. Diagnostic and treatment algorithms are continually evolving, and gene therapy is emerging as a promising future option, offering hope beyond current treatments like liver transplantation and medical therapy^[5-7]^.

According to existing literature, effective treatment options for PFIC-3 remain limited, making the discovery of new approaches and ongoing research critical. Here, we report a novel PFIC-3 variant. To our knowledge, this variant has not been previously reported in the literature or in large population databases such as the genome aggregation database (gnomAD: https://gnomad.broadinstitute.org). We also conducted a literature review of published ABCB4 mutations available on PubMed.

## Case presentation

A 16-year-old female patient was admitted to the hospital in November 2019 due to diffuse abdominal pain, accompanied by mildly elevated serum liver enzyme levels. She showed no signs of overall liver dysfunction, so no further investigation was conducted, and she was managed conservatively.

The patient had no history of hepatotoxic drug use, exposure to toxins, or alcohol abuse. There were no identifiable factors that triggered the onset of the disease, nor was there a similar episode in her childhood. Her parents, who are first-degree cousins, were healthy. There was a family history of bile duct stones in her maternal grandmother and liver cirrhosis in her paternal uncle, who had a history of alcohol intake (amounts unknown) and died at age 30. All her siblings were healthy.

**Figure 1.**
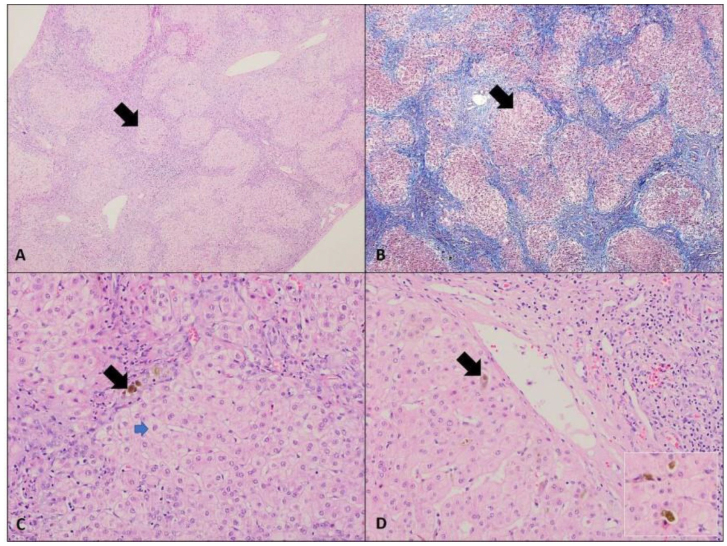
Biliary-pattern cirrhosis in PFIC-3. (A) Disrupted hepatic architecture with presence of portal-portal bridging fibrosis and irregular/jigsaw puzzle-shaped regenerating hepatocytic nodules (arrow) (H&E, 4X). (B) Masson trichrome highlights the fibrosis (blue color) as well as the pattern of cirrhosis (4X). (C) Occasional bile plugs in portal bile ductules are noted (black arrow). Frequent hepatocellular changes including clearing and feathery degeneration is noted (blue arrow) (20X). (D) Focal hepatocellular and canalicular bile is noted (arrow and insert) (20X).

In July 2021, 2 years later, the patient was admitted again with fever, jaundice, and right upper quadrant abdominal pain. An abdominal ultrasound revealed a distended, non-inflamed gallbladder containing sludge, a dilated common bile duct, and hepatosplenomegaly. Endoscopic retrograde cholangiopancreatography (ERCP) was performed, showing no abnormalities in the intrahepatic biliary system, but a stone was found in the cystic duct, and a stent was inserted. The patient was then scheduled for an elective laparoscopic cholecystectomy. During the procedure, chronic cholecystitis with adhesions, a normal common bile duct, and a diseased, nodular liver were discovered. A liver biopsy revealed advanced fibrosis with hepatic nodules, significant architectural distortion, and no detectable iron staining (Fig. 1).

A comprehensive evaluation for liver fibrosis revealed thrombocytopenia, hyperammonemia, direct hyperbilirubinemia, and elevated levels of alanine aminotransferase, aspartate aminotransferase, GGT, and alkaline phosphatase. The coagulation profile, albumin level, and ferritin level were all within normal ranges. Tests for viral hepatitis (hepatitis B surface antigen, hepatitis C virus antibodies, and HB core antibodies) were negative. Additional tests for antinuclear, antimitochondrial, and anti-tissue transglutaminase antibodies, among others, were also negative. Alpha-fetoprotein, ceruloplasmin, and cortisol were within normal limits, and there was no evidence of Kayser-Fleischer rings on ophthalmic examination. The echocardiogram showed normal results except for trace pericardial effusion and tricuspid regurgitation.

An upper endoscopy was performed as part of annual surveillance to rule out esophageal and gastric varices secondary to liver disease, revealing only gastritis, which was treated with proton pump inhibitors (PPIs). On follow-up visits, laboratory results remained elevated. Repeated ultrasound showed moderate abdominopelvic ascites and prominent portal and splenic veins.

Given the patient’s medical history, hepatosplenomegaly, portal hypertension, laboratory findings, and family history of liver disease, we pursued genetic testing. After obtaining consent, a whole blood sample was collected from the patient in December 2021. Genomic DNA was extracted, and DNA sequences were captured using hybridization probes. Sequencing was performed using Illumina’s Reversible Dye Terminator (RDT) platform, NovaSeq 6000, with 150 by 150 bp paired-end reads.

**Figure 2.**
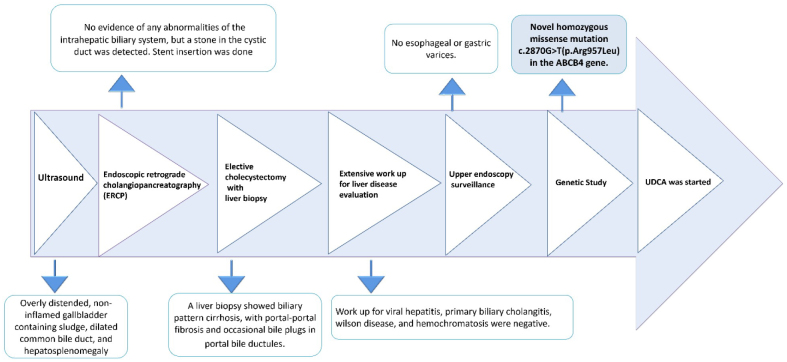
Clinical course of patient diagnoses: the figure illustrates the timeline of diagnostic tests and procedures for the patient.

Sequence analysis of common genes responsible for cholestasis revealed a novel homozygous c.2870G > T mutation in the ABCB4 gene, resulting in the amino acid substitution p. Arg957Leu. A manually created illustration of the ABCB4 gene and MDR3 protein was provided to depict the site and nature of this novel mutation (Fig. 2).

The clinical significance of this variant is currently uncertain due to the lack of conclusive functional and genetic evidence, and it is not listed in the ClinVar database. Sequencing both parents is necessary to confirm their carrier status and ensure that both alleles were sequenced in the patient, but this was not pursued due to financial constraints. Automated tools, PolyPhen-2 and SIFT, predicted the novel mutation p. Arg957Leu as damaging. FATHMM, another tool for differentiating between pathogenic and neutral variants, also classified this mutation as pathogenic.

PFIC-3 was diagnosed provisionally, and the patient was prescribed ursodeoxycholic acid (UDCA) and vitamins, which led to improvements in overall health and jaundice (Fig. 2). Notably, during follow-up visits, the patient’s liver function had fully normalized, and she remained in complete remission.

## Literature review design

PubMed was searched for published cases of PFIC-3 and ABCB4 mutation. We used search strategy for relevant cases; ABCB4 OR ABCB4 gene OR ABCB4 variant OR MDR3, case report OR case series OR case, and progressive familial intrahepatic cholestasis 3 OR PFIC3. We made a comprehensive table for review of retrieved cases (Table 2 and Supplementary Digital Content, Table 1, http://links.lww.com/MS9/A707)

**Table 1:** Dynamic changes of liver tests of the patient

	AST	ALT	ALP	GGT	Total B	Direct B
	(0.7-55 U/L)	(0.8-48 U/L)	(44-147IU/L)	(0-30IU/L)	(0.1-1.2 mg/dL)	(Up to 0.3 mg/dL)
2 years previously	96	179	570	NA	2.8	–
After cholecystectomy and ERCP	117	79	114	NA	2.6	1.6
Two months after cholecystectomy	102	NA	226	60	2.88	2.4
UDCA was started						
After 4 months of UDCA treatment	71	57	213	39	1.3	0.5
Now	50	37	–	–	0.7	0.4

ALP, alkaline phosphatase; ALT, alanine aminotransferase; AST, aspartate aminotransferase; B, Bilirubin; ERCP, Endoscopic retrograde cholangiopancreatography; GGT, gamma-glutamyl transferase; UDCA, ursodeoxycholic acid

**Table 2:** Summary of the reported PFIC-3 related ABCB4 mutations in the literature review.

Study ID	Missense, splicing, or deletion?	Novel mutation?	Heterozygous or homozygous	ABCB4 mutation	Need for transplant	Response to UDCA	Mortality
**Lulecioglu 2024^[18]^**	Missense	No	Homozygous	c.2950G > A, p. Ala984Thr	NA	NA	No
**Cao 2024^[14]^**	**Pt 1:** Missense/Missense/Missense	Yes	**Pt 1:** Compound Heterozygous	**Pt 1**: c.2570C>T p.T857I/ c.2212A>T p.I738F/ c.1694C>GpT56	**Pt 6, 10, 14**	**Pt 1, 4, 5, 7, 16** were stable on UDCA/	Yes (**Pt 20**)
	**Pt 2:** Missense/Missense/Missense		**Pt 2:** Compound Heterozygous	**Pt 2**: c.2570C>T p.T857I/c.2212A>T p.I738F/c.1694C>G p.T565R			
			**Pt 3:** Homozygous	**Pt 3:** c.1241G>T p.G414V/c.1241G>T p.G414V			
	**Pt 3:** Missense/Missense		**Pt 4:** Compound Heterozygous			**Pt 2, 3, 11, 12** progressed	
	**Pt 4:** Missense/Nonsense		**Pt 5:** Compound Heterozygous	**Pt 4**: c.3152T>C p.V1051A/**c.589C≥T p.Q197X**			
	**Pt 5:** Missense/Missense		**Pt 6:** Homozygous	**Pt 5: c.1058G≥A p.C353Y/ c.956G≥T p.G319V**			
	**Pt 6:** Missense/Missense		**Pt 7:** Compound Heterozygous	**Pt 6**: c.1195G>C p.V399L/c.1195G>C p.V399LPt			
	**Pt 7:** Missense/Missense		**Pt 10**: Compound Heterozygous	**Pt 7: c.473T≥A p.L158Q/ c.164T≥C p.L55S**			
	**Pt 10**: Splicing/Missense		**Pt 11**: Compound Heterozygous	**Pt 10: c.2493G≥C splicing mutation/ c.1230±1G≥ A p.R831S**			
	**Pt 11**: Missense/Missense		**Pt 12**: Homozygous	**Pt 11**: c.140G>A p.R47Q/ **c.1150G≥C p.G384R**			
	**Pt 12**: Missense/Missense		**Pt 14**: Compound Heterozygous	**Pt 12**: c.1195G>C p.V399L /c.1195G>C p.V399L			
	**Pt 14**: Duplicate/Missense		**Pt 16**: Compound Heterozygous	**Pt 14** c.1015dup p.S339Ffs*17/ **c.2914G≥A p.D972N**			
	**Pt 16**: Splicing/Missense		**Pt 20**: Compound Heterozygous	**Pt 16: c.2317-5A≥G Splicing mutation/ c.1650C≥A p.N550K**			
	**Pt 20**: Deletion/Deletion with			Pt 20: c.3269_3271del p.Ala1090del/c.3252_3264del p.F1085Wfs*57			
	frameshift						
**Cheng 2023^[19]^**	Missense	NA	Heterozygous	c.2318G>T, p. Gly773Val	NA	Yes	No
**Shankar 2023^[20]^**	Missense	Yes	Homozygous	**c.208G ≥ A, p. Thr1077Met**	Yes	NA	No
**Shen 2023^[21]^**	Missense and Nonsense	Yes	Compound Heterozygous	Pt 1: **c.646C ≥ T, p.L216F/c.927T ≥ A, p.Y.309X**	NA	Yes	No
	NA/missense/missense		Compound Heterozygous	Pt 2: **c.2784-1G ≥ A in intron 22 region/** c.646C > T p.L216F/c.504C > T p. N168N		No	Yes
**Ruiqi 2023^[22]^**	Nonsense	NA	Heterozygous	c.716 C>A p. Ser239 Ter, 1048	No	Yes	No
**Gonzales 2023^[23]^**	**Pt 1:** Duplicate with nonsense	NA	**Pt 1:** Homozygous	**Pt 1:** c.2202dupG; p. (Ile735AspfsX10)	**Pt 1:** Yes	**Pt 1:** NA	**Pt 1:** Yes
	**Pt 2:** Duplicate with nonsense		**Pt 2:** Homozygous	**Pt 2:** c.2202dupG; p. (Ile735AspfsX10)	**Pt 2:** Yes	**Pt2**: NA	**Pt 2:** No
	**Pt 3:** Duplicate with nonsense		**Pt 3:** Homozygous	**Pt 3:** c.2202dupG; p. (Ile735AspfsX10)	**Pt 3**: Yes	**Pt 3**: No	**Pt 3:** No
	**Pt 4**: Deletion with nonsense		**Pt 4:** Homozygous	**Pt 4**: c.1712delT; p. (Val571AspfsX16)	**Pt 4:** Yes	**Pt 4**: No	**Pt 4:** No
	**Pt 5:** Nonsense		**Pt 5:** Homozygous	**Pt 5:** c.1906 C>T; p. (Gln636X)	**Pt 5:** Yes	**Pt 5:** NA	**Pt 5**: No
	**Pt 6:** Nonsense		**Pt 6:** Homozygous	**Pt 6**: c.1906 C>T; p. (Gln636X)	**Pt 6:** Yes	**Pt 6:** No	**Pt 6:** No
	**Pt 7:** Nonsense		**Pt 7:** Homozygous	**Pt 7:** c.2869C>T; p. (Arg957X)	**Pt 7:** Yes	**Pt 7:** No	**Pt 7:** Yes
	**Pt 8:** NA		**Pt 8:** Homozygous	**Pt 8:** c.79A>G; p.(?)	**Pt 8:** Yes	**Pt 8:** No	**Pt 8:** No
	**Pt 9:** Deletion with nonsense		**Pt 9:** Homozygous	**Pt 9:** c.1712delT; p. (Val571AspfsX16)	**Pt 9:** Yes	**Pt 9:** No	**Pt 9:** No
	**Pt 10**: Deletion with nonsense/ nonsense		**Pt 10**: Compound Heterozygous	**Pt 10:** c.1712delT; p. (Val571AspfsX16)/c.139C>T; p. (Arg47X)	**Pt 10**: Yes	**Pt 10**: No	**Pt 10**: No
			**Pt 11**: Homozygous	**Pt 11**: c.3081+5G>C; p.(?)	**Pt 11**: Yes	**Pt 11**: No	**Pt 11**: No
	**Pt 11**: NA		**Pt 12**: Compound Heterozygous	**Pt 12:** c.2132T>C; p. (Phe711Ser)/c.1006-28_1435del; (p.?)	**Pt 12**: No	**Pt 12**: Yes	**Pt 12:** No
	**Pt 12**: Missense/ Deletion		**Pt 13**: Compound Heterozygous	**Pt 13:** c.1270A>G; p. (Thr424Ala)/c.2925-10_2925-9insC; p.(?)	**Pt 13**: Yes	**Pt 13**: NA	**Pt 13**: No
					**Pt 14**: No	**Pt 14**: Yes	**Pt 14**; No
	**Pt 13**: Missense/Insertion		**Pt 14**: Compound Heterozygous	**Pt 14:** c.344+2T>C; p.(?)/c.2800G>A; p. (Ala934Thr)	**Pt 15**: Yes	**Pt 15**: Partial	**Pt 15**: No
	**Pt 14**: NA/missense		**Pt 15**: Compound Heterozygous	**Pt 15:** c.2943-2952del; p. (Phe982TrpfsX3) /c.2947G>A; p. (Gly983Ser)	**Pt 16**: Yes	**Pt 16**: No	**Pt 16:** No
	**Pt 15**: Deletion with nonsense/ missense		**Pt 16**: Compound Heterozygous		**Pt 17**: No	**Pt 17**: Partial	**Pt 17:** No
			**Pt 17**: Compound Heterozygous	**Pt 16**: c.412T>C; p. (Trp138Arg)/c.3486+1063ins148bp;p.(?)p. Lys1163LeufsX4	**Pt 18**: No	**Pt 18**: Yes	**Pt 18:** No
			**Pt 18**: Compound Heterozygous		**Pt 19**: No	**Pt 19**: Partial	**Pt 19:** No
	**Pt 16**: Missense/ Insertion with nonsense		**Pt 19**: Homozygous	**Pt 17**: c.2T>C; p.(?)/c.2876G>T; p. (Gly959Val)	**Pt 20**: Yes	**Pt 20**: NA	**Pt 20**: Yes
			**Pt 20**: Homozygous	**Pt 18**: c.2800G>A; p. (Ala934Thr)/c.2924+1G>A; p.(?)	**Pt 21**: Yes	**Pt 21**: NA	**Pt 21**: No
	**Pt 17**: NA/Missense		**Pt 21**: Homozygous	**Pt 19**: c.2800G>A; p. (Ala934Thr)	**Pt 22**: Yes	**Pt 22**: NA	**Pt 22**: No
					**Pt 23**: No	**Pt 23**: Partial	**Pt 23**: No
	**Pt 18: M**issense/NA		**Pt 22**: Homozygous	**Pt 20**: c.2860G>A; p. (Gly954Ser)	**Pt 24**: No	**Pt 24**: Yes	**Pt 24:** No
	**Pt 19**: Missense		**Pt 23**: Homozygous	**Pt 21**: c.1184A>G; p. (Glu395Gly)	**Pt 25**: No	**Pt 25**: Yes	**Pt 25:** No
	**Pt 20**: Missense		**Pt 24**: Compound Heterozygous	**Pt 22**: c.1667T>G; p. (Leu556Arg)	**Pt 26**: No	**Pt 26**: Yes	**Pt 26:** No
			**Pt 25**: Compound Heterozygous	**Pt 23**: c.2564A>T; p. (Gln855Leu)	**Pt 27**: No	**Pt 27**: Yes	**Pt 27:** No
	**Pt 21**: Missense		**Pt 26**: Homozygous	**Pt 24**: c.140G>A; p. (Arg47Gln)/c.2317G>C; p. (Gly773Arg)	**Pt 28**: No	**Pt 28**: Yes	**Pt 28:** No
	**Pt 22**: Missense		**Pt 27**: Compound Heterozygous	**Pt 25**: c.1072G>C; p. (Ala358Pro) /c.1691A>G; p. (Asp564Gly)	**Pt 29**: No	**Pt 29**: Partial	**Pt 29**: No
	**Pt 23**: Missense		**Pt 28**: Homozygous	**Pt 26**: c.2860G>A; p. (Gly954Ser)	**Pt 30**: Yes	**Pt 30**: No	**Pt 30:** No
	**Pt 24**: Missense/ Missense		**Pt 29**: Homozygous	**Pt 27**: c.1373A>C; p. (Gln458Pro)/c.2800G>A; p. (Ala934Thr)	**Pt 31**: No	**Pt 31**: Yes	**Pt 31**: No
	**Pt 25**: Missense /Missense		**Pt 30**: Homozygous	**Pt 28**: c.1880 T>C; p. (Leu627Pro)	**Pt 32**: No	**Pt 32**: Yes	**Pt 32**: No
	**Pt 26**: Missense		**Pt 31**: Compound Heterozygous	**Pt 29**: c.1880 T>C; p. (Leu627Pro)	**Pt 33**: No	**Pt 33**: Yes	**Pt 33:** No
	**Pt 27**: Missense/ Missense		**Pt 32**: Compound Heterozygous	**Pt 30**: c.3226A>T; p. (Ser1076Cys)	**Pt 34**: No	Pt 34: Yes	**Pt 34:** No
	**Pt 28**: Missense		**Pt 33**: Homozygous	**Pt 31**: c.2800 G>A; p. (Ala934Thr)/c.1348_1353del; p.	**Pt 35**: No	**Pt 35**: Yes	**Pt 35**: No
	**Pt 29**: Missense		**Pt 34**: Compound Heterozygous	(Glu450_Gly451del)	**Pt 36**: No	**Pt 36**: Yes	**Pt 36**: No
	**Pt 30**: Missense		**Pt 35**: Compound Heterozygous	**Pt 32**: c.2800G>A; p. (Ala934Thr)/c.2932T>C; p. (Ser978Pro)	**Pt 37**: No	**Pt 37**: Yes	**Pt 37:** No
	**Pt 31**: Missense/ Deletion		**Pt 36**: Compound Heterozygous	**Pt 33**: c.524C>A; p. (Thr175Lys)	**Pt 38**: No	**Pt 38**: Yes	**Pt 38:** No
	**Pt 32**: Missense/ Missense		**Pt 37**: Homozygous	**Pt 34**: c.431G>A; p. (Arg144Gln)/c.3233T>A; p. (Val1078Glu)			
	**Pt 33**: Missense		**Pt 38**: Homozygous	**Pt 35**: c.1295G>A; p. (Gly432Asp)/Allele 2: c.959 C>T; p. (Ser320Phe)			
	**Pt 34**: Missense/ Missense			**Pt 36**: c.140G>A; p. (Arg47Gln)/c.1296_1301del; p. (Cys433_Gly434del)			
	**Pt 35**: Missense/ Missense						
	**Pt 36**: Missense/ Deletion			**Pt 37**: c.1529A>C; p. (Asn510Thr)			
	**Pt 37**: Missense			**Pt 38**: c.1529A>C; p. (Asn510Thr)			
	**Pt 38**: Missense						

**Qiao 2023^[24]^**	Missense/ deletion	Yes	Heterozygous	**c.2696C≥G (p. Ala899Gly), exon 22 ch7 A≥G/c.3487_3571del.**	No	Yes	No
**Zampaglione 2023^[25]^**	Missense	No	Homozygous	c.572A.G; p. (Lys191Arg)	Yes	Initially yes. One year later needed liver transplantation due to encephalopathy	No
**Cheng 2022^[26]^**	**Pt1**: Hybrid splicing	Yes	Hybrid mutations	**Pt 1: c.1560±2T ≥ A**	NA	NA	NA
	**Pt2, Pt3:** Hybrid missense			**Pt 2:** c.2177C > T (p. Pro726Leu),			
	**Pt4**: Missense			**Pt 3: c.1756G ≥ T (p. Val586Leu**), c.2177C > T (p. Pro726Leu),			
				**Pt 4: c.2471T ≥ C (p. Val824Ala)**			
**Liu 2022^[27]^**	Missense/Missense	Yes	Heterozygous	**c.2950C≥T, p.A984V (exon 24)/ c.667A≥G; p.I223V (exon 7)**	No	Yes	No
**Mehta 2022^[28]^**	NA	NA	Heterozygous	c.1529 A > G, Exon 13/c.370G>C, Exon 6	Yes, both patients	NA	Yes, One of them
				The other patient: NA			
**Zhu 2022^[29]^**	Missense/Missense	One mutation is novel.	Compound Heterozygous	c.959C > T (p.S320F)/**Novel: c.1429C≥A (p.Q477K)**	Yes	No	No
**Alasmari 2022^[30]^**	NA	No	Homozygous	c.2906G>A	Yes	No	No
**Bai 2021^[31]^**	Missense/Missense	Yes	Compound Heterozygous	**c.T2525C (p.L842P)/c. T3152C (p.V1051A)**	No	Yes	No
**Chiou 2021^[32]^**	Missense	No	Combined Heterozygous	ABCB4: c. 1954A>G (p.R652G)/ ATP8B1 (c. 2855G>A (p. R952Q)	Yes	NA	No
**Sinha 2021^[33]^**	Missense/ Duplicate with nonsense	One mutation is novel.	Compound Heterozygous	**Pt 1:** c.959C>T;(Ser320Phe)/**c.2301dupT, p. (Thr768TyrfsTer26)**	Yes	**Pt 1/2:**	No
	Missense/ Duplicate with nonsense			**Pt 2:** c.959C>T, p. (Ser320Phe)/**c.2301dupT, p(Thr768TyrfsTer26)**	(all patients)	NA	
	Missense/ Duplicate with nonsense			**Pt 3:** c.959C >T; p. (Ser320Phe)/c.**2301dupT; p. (Thr768TyrfsTer26)**		**Pt 3:** No	

**Lipinski 2020^[34]^**	Pt 1: Missense/Missense	Three Novel	Compound Heterozygous	**Pt 1:** c.2149T>A, p. Cys717Ser/c.1745G>A, p. Arg582Gln	Yes (Pt 1 and Pt 2)	**Pt:3** Yes	No
	Pt 2: Missense/Missense	Mutations		**Pt 2: c.3524T≥A, p. Leu1175His/**c.3524T>A, p. Leu1175His		**Pt 4:** No	
	Pt 3: Missense/ NA			**Pt 3:** c.959C>T, p. Ser320Phe/c.1119+1G>A, p.?			
	Pt 4: Missense/NA			**Pt 4: c.902T≥A, p. Met301Lys/c.3279±1G≥A, p.?**			

**Goubran 2020^[35]^**	Missense/Missense	One mutation is novel.	Compound Heterozygous	c.3227G > A (p.S1076N)/ **c.3431T ≥ C (p.I1144T)**	Yes	NA (before transplant)	No
**Saleem 2020^[36]^**	Missense	Yes	Homozygous	**c.1195G≥C: p.V399L. (exon 11)**	NA	NA	No
**Sticova 2020^[37]^**	Missense/NA	Yes	Heterozygous	**c.833±1G≥T, p. Ile600Phe // c.1798T≥A**	Yes	NA	No
**Yavas 2020^[38]^**	NA/NA	Yes	Compound heterozygous	**NM_018849.2: c.1565≥C and NM_018849.2: c.3859T≥A.**	No	No	No
**Wu 2020^[39]^**	Missense/Missense	One mutation is novel.	Heterozygous	**c.2137G≥A; p.V713M (Exon 17)**/c.504C>T; p.N168N (Exon 6)	Yes	No	No
**Zhang 2020^[40]^**	**Pt 1**: Insertion with nonsense/Insertion and deletion	Yes, 6 novel mutations	Compound Heterozygous	**Pt 1: c.2489insA ≥ p. T830NfsX11/c.3139_3141delGCAinsCC ≥ p. A1047PfsX8**	No	NA	**Pt 1/ 2:** NA
			Compound Heterozygous				**Pt 3:** No
	**Pt 2:** Missense/Missense		Homozygous	**Pt 2: c.955G≥C ≥ p.G319R/c.3220G≥A ≥ p.G1074R**			
	**Pt 3:** Missense		Compound Heterozygous	**Pt 3:** c.1436C>T > p.P479L			**Pt 4:** Yes
	**Pt 4:** Deletion with nonsense/ Missense			**Pt 4: c.3143delA ≥ p. N1048TfsX/c.3139G≥C ≥ p. A1047P**			
**Masi 2019^[41]^**	Missense/NA	NA	Double Heterozygous	c.1954A > G, p. Arg652Gly /c.711A > T, p. Ile237	No	Yes	No
**Zhang 2019^[42]^**	Splicing	No	NA	IVS13+6G>A/G	No	Yes	No
**Tan 2018^[43]^**	Missense	No	Heterozygous	ex13 c.1531G > A (p.A511T)	No	Yes	No
**Xiang 2017^[44]^**	Missense/Missense	One novel mutation	Heterozygous	**c.2362C≥T p. Arg788Trp/**c.1798A>G p. Ile600Val.	Yes	Yes, after liver transplantation	No
**Oliveria 2016^[45]^**	Nonsense/Missense	Yes	Compound Heterozygous	c.874A≥T (p. Lys292*)/c.3680T≥C. (p. Ile1227Thr)	NA	No	NA
**Wang Z 2016^[46]^**	Missense/ synonymous	Yes	NA	**c.1634G ≥ A, p.R545H**/synonymous mutation (c.504C > T, p.N168N)	Yes, but her daughter no	Mother: No Daughter: yes	No

**Giovannoni 2015^[47]^**	**Pt 1:** Nonsense.	One novel mutation	Homozygous	c.1783C>T p.R595X	**Pt 1:** Yes	NA	**Pt 1 and 2**:
	**Pt 2:** Nonsense/Deletion and insertion		Compound Heterozygous Compound Heterozygous	c.1783C>T p.R595X/c.937_992ins/del6	**Pt 2:** Yes		No
	**Pt 3:** Missense/Missense			**c.1442T≥G p.L481R**/c.1954A>G p. R652G	**Pt 3:** No		**Pt 3**: NA
**Frider 2015^[16]^**	Missense/Missense	Yes	Compound Heterozygous	**c.140G ≥ A p.R47Q/c.245C ≥ A, p.T82N**	No	Yes	No
	Missense		Heterozygous	**c.245C≥A (p.T82N)**			
**Sun 2015^[48]^**	All missense except this Exon 9 c.874 A>T (p.K292X) nonsense	Yes, one of them	Heterozygous	Exon 4 c.175 C>T (p.L59L)	NA	NA	NA
				Exon 6 c.504 C>T (p.N168N)			
				Exon 8 c.711 A>T (p.I237I)/ Exon 9 c.874 A>T (p.K292X)			
				**Exon 15 c.1804G≥T (p.G602W)**			
**Gordo-Gilart 2014^[49]^**	**Pt 1:** Nonsense/Splicing.	NA	**Pt 1:** Compound Heterozygous	**Pt 1:** c.3559C>T p.R1187X/ c.3633+2T>A Splicing defect	**Pt 1/2/3/4**	**Pt 5/6** Yes	No
	**Pt 2/3/4/5/6:** Missense		**Pt 2/3:** Homozygous	**Pt 2:** c.202G>A p.G68R	Yes	**Pt 1/2/3:** Partial	
			**Pt 4/5**: Compound Heterozygous	**Pt 3:** c.202G>A p.G68R	**Pt 5/6** No		
			**Pt 6:** Heterozygous	**Pt 4:** c.1375G>C p.D459H/ c.1436C>T p.P479L		**Pt 4:** No	
				**Pt 5:** c.602C>T p.T201M/ c.3352G>A p.E1118K			
				**Pt 6:** c.2932T>C p. S978P			
**Dzagania 2012^[50]^**	Missense	Yes	Homozygous	**c.3691C≥T, p.H1231Y**	No	Yes	No
**Poupon 2009^[51]^**	Missense/Missense	No	Heterozygous non-synonymous variants	**Pt 1 and Pt 2**: c.2858C 4A; p. Ala953Asp/ c.959C 4T; p. Ser320Phe	Yes	**Pt 1:** No	No
						**Pt 2:** NA	

Bold with line below mutation indicates the novel mutation. NA, not applicable; Pt, patient; VOUS, variant of unknown significance.

## Discussion

PFIC3 is an autosomal recessive disease characterized by early cholestasis, jaundice, and progressive liver damage. A survey showed that the mean incidence of PFIC3 is 1 in 500 000 people^[8]^. Expanding the understanding of PFIC3 is important as it has the potential to enhance knowledge of this disease. Our study reported a young female patient with a c.2870G > T (p. Arg957Leu) mutation in the ABCB4 gene who responded dramatically to UDCA without requiring a liver transplant. To our knowledge, this mutation has not been previously reported in the literature.

We reviewed 108 cases from 36 published studies, all with different mutations in the PFIC3 gene (Table 2 and Supplementary Digital Content, Table 1, http://links.lww.com/MS9/A707). Of these, 104 were diagnosed solely as PFIC3, one case was diagnosed as PFIC3 with cirrhosis and systemic amyloidosis, another as PFIC1 & PFIC3 with myotonic dystrophy, one as PFIC3 and multiple sclerosis, and one as PFIC3 and biliary atresia. The age of patients ranged from 1 day to 45 years. Of these, 53 (nearly 50%) were females. The most common presenting signs and symptoms were abdominal pain, jaundice, pruritus, and hepatosplenomegaly. Elevated GGT levels were observed in 105 cases (97.22%), while 10 cases had normal MDR3. Most mutations were compound heterozygous (n = 103), followed by homozygous mutations (n = 38) and heterozygous mutations (n = 24). Missense mutations were the most common type (n = 118), and 56 novel mutations were identified.

Fifty out of 108 cases responded to UDCA. Of the 21 patients who did not respond to UDCA, 9 had compound mutations (42.85%), 9 had homozygous mutations (42.85%), and 11 had missense mutations (52.38%). Liver transplantation was required in 46 cases (42.6%), and 7 patients died. Most of those needing liver transplantation had compound mutations (n = 17, 36.95%), homozygous mutations (n = 21, 45.65%), and missense mutations (n = 30, 65.2%). Among the mortality cases (n = 7), 3 had missense mutations (42.85%), 3 had compound mutations (42.85%), and 4 had homozygous mutations (57%). Among patients with low or absent MDR3 levels (n = 35), compound mutations were found in 15 cases (42.85%), homozygous mutations in 12 cases (34.28%), and missense mutations in 26 cases (74.28%). Of these, 16 (45.71%) required liver transplantation, while 15 (42.85%) responded to UDCA.

PFIC3 typically presents with jaundice, pruritus, pale stools, and hepatosplenomegaly^[9]^. These symptoms can resemble other cholestatic conditions, making early diagnosis challenging. If left untreated, PFIC3 can lead to serious complications such as liver failure, hepatic encephalopathy, and gastrointestinal bleeding^[9]^. In our case, the primary concern was that the diagnosis was confirmed only after extensive resource use, with a prolonged period during which the patient initially presented with symptoms, but her abnormal liver enzymes were not investigated. The patient returned to the hospital a year later, during which time numerous health deterioration scenarios could have occurred.

The diagnosis of PFIC3 relies on a multisystemic approach, including history and physical examination, laboratory tests, imaging, histopathology with immunohistochemistry, and genetic studies^[9]^. PFIC3 is distinguished by elevated GGT levels, in contrast to PFIC1 and PFIC2^[3]^. Abdominal ultrasound is usually the first imaging modality used and often shows evidence of cholestasis, portal hypertension, and cirrhosis^[3]^. Liver histopathology is a key diagnostic tool and allows immunostaining to be performed. Portal fibrosis and ductular proliferation with a mixed inflammatory infiltrate are often found^[3,9]^. Immunohistochemistry may show absent MDR3 staining; however, normal staining does not exclude MDR3 dysfunction. If PFIC3 is suspected or other common cholestatic diseases are ruled out, genetic testing is performed.

Sequence analysis of the ABCB4 gene typically reveals homozygous and compound heterozygous mutations^[3,10]^. Heterozygous mutations may result in less severe diseases, such as ICP, contraceptive-induced cholestasis, low phospholipid-associated cholelithiasis, transient neonatal cholestasis, drug-induced liver injury, or adult idiopathic cirrhosis^[9-12]^. This is because mutant proteins may negatively impact protein function or production^[9]^.

ABCB4 mutations are classified into different types: nonsense variations (I), or missense variations that affect the maturation (II), activity (III), or stability (IV) of the MDR3 protein, and mutations without detectable defects (V)^[13]^. These mutations can result in functional impairment, truncated non-functional proteins, or even complete loss of protein production^[3]^. Over 500 ABCB4 mutations have been discovered, with the majority being missense mutations^[3]^. Fortunately, missense mutations tend to present later in life, with milder disease, slower progression, and better treatment response^[14]^. This is because missense mutations result in partially functional transport activity^[9]^. Additionally, missense mutations are often associated with normal to faint MDR3 immunohistochemical staining^[12]^.

There are multiple methods for managing PFIC3 patients, including (1) medications such as UDCA, fat-soluble vitamins, cholestyramine, and rifampicin, (2) nutritional support, (3) surgical interventions, and (4) liver transplantation. Recently, gene therapy has been developed and is still under study^[15]^.

UDCA remains the most effective medical treatment. In a study by Frider *et al*, long-term use of UDCA in MDR3 heterozygotes with residual protein expression was associated with the reversal of fibrosis^[16]^. UDCA is a safe drug, effective in reversing the hepatotoxicity of accumulated bile acids, controlling bile acid distribution, reducing cholesterol levels, and maintaining mitochondrial integrity. Studies have shown that treating cholestasis with doses of 10 to 30 mg/kg per day is effective^[17]^.

Within a few months of treatment, our patient’s liver enzyme and bilirubin levels had normalized. According to a study by Frider *et al*, about half of the patients who received oral UDCA therapy showed a positive response. It restored normal liver function tests and prevented progression to cirrhosis. In contrast, non-responders required liver transplantation. These variations in response were linked to specific ABCB4 mutations. Missense mutations were associated with milder disease, whereas mutations that completely prevented MDR3 expression led to severe liver damage resistant to UDCA therapy^[16]^.

The uniqueness of this case lies in the discovery of a novel **ABCB4** mutation (c.2870G > T, p. Arg957Leu), which has not been previously documented. While PFIC3 patients with other **ABCB4** mutations have varied responses to UDCA, this specific mutation resulted in a complete biochemical remission without the need for liver transplantation. This highlights the clinical importance of identifying new genetic mutations, as they can offer insights into the variability of disease progression and treatment outcomes in PFIC3. Our case adds to the growing body of evidence that genotype-specific approaches may enhance patient care and optimize treatment strategies for this rare disease.

## Conclusions

This case underscores the importance of timely diagnosis and genetic testing in managing PFIC3. Identifying ABCB4 mutations can guide treatment, as shown by our patient’s response to UDCA, avoiding liver transplantation. Variability in PFIC3 presentation and treatment outcomes, as demonstrated by our review of 108 cases, highlights the need for personalized approaches. While UDCA remains an effective option for some, liver transplantation is still required in severe cases. Ongoing research into genotype-phenotype correlations and emerging therapies like gene therapy will be crucial for improving long-term outcomes in PFIC3 management.

## Data Availability

The datasets used and/or analyzed in this study are available from the corresponding author on reasonable request.
